# Mitigating Harmful Cyanobacterial Blooms in a Human- and Climatically-Impacted World

**DOI:** 10.3390/life4040988

**Published:** 2014-12-15

**Authors:** Hans W. Paerl

**Affiliations:** Institute of Marine Sciences, the University of North Carolina at Chapel Hill, 3431 Arendell Street, Morehead City, NC 28557, USA; E-Mail: hpaerl@email.unc.edu; Tel.: +1-252-726-6841(ext. 133)

**Keywords:** harmful cyanobacteria, nitrogen, phosphorus, water quality management, mitigation, climate change

## Abstract

Bloom-forming harmful cyanobacteria (CyanoHABs) are harmful from environmental, ecological and human health perspectives by outcompeting beneficial phytoplankton, creating low oxygen conditions (hypoxia, anoxia), and by producing cyanotoxins. Cyanobacterial genera exhibit optimal growth rates and bloom potentials at relatively high water temperatures; hence, global warming plays a key role in their expansion and persistence. CyanoHABs are regulated by synergistic effects of nutrient (nitrogen:N and phosphorus:P) supplies, light, temperature, vertical stratification, water residence times, and biotic interactions. In most instances, nutrient control strategies should focus on reducing both N and P inputs. Strategies based on physical, chemical (nutrient) and biological manipulations can be effective in reducing CyanoHABs; however, these strategies are largely confined to relatively small systems, and some are prone to ecological and environmental drawbacks, including enhancing release of cyanotoxins, disruption of planktonic and benthic communities and fisheries habitat. All strategies should consider and be adaptive to climatic variability and change in order to be effective for long-term control of CyanoHABs. Rising temperatures and greater hydrologic variability will increase growth rates and alter critical nutrient thresholds for CyanoHAB development; thus, nutrient reductions for bloom control may need to be more aggressively pursued in response to climatic changes globally.

## 1. Introduction

Cyanobacteria are the Earth’s oldest known prokaryotic oxygenic phototrophs, having appeared over 2.5 billion years ago [1]. They have witnessed major biogeochemical and climatic changes, including extreme swings in irradiance and temperature, rising oxygen levels (largely due to their own photosynthetic activities) and altered chemical composition of the Earth’s atmosphere [1]. They have experienced periods of high and low nutrient (N, P, minor elements) abundance, and a great deal of variability in climatic conditions, including extremely wet and dry periods, combined with major oscillations in the Earth’s surface temperature, geophysical processes such as volcanism and climatic shifts due to continental drift. These events have radically altered cyanobacterial habitats and exerted eco-physiological stresses over a wide range of spatial and temporal scales.

A long evolutionary history has provided cyanobacteria with an extensive ecophysiological “playbook” to deal with environmental extremes and constraints [2,3]. This, in combination with plenty of time for structural and functional diversification, has enabled cyanobacteria to occupy extremely broad geographic habitats, ranging from polar to tropical regions [2]. They can be found in virtually all terrestrial and aquatic ecosystems, ranging from deserts to tropical rain forests, to alpine and subsurface soils, and from the ultraoligotrophic open ocean to hypereutrophic lakes [2,3]. Over geological and biological time scales, cyanobacteria reveal a remarkable ability to counter extreme geochemical and climatic conditions and thrive under them.

**Figure 1:**
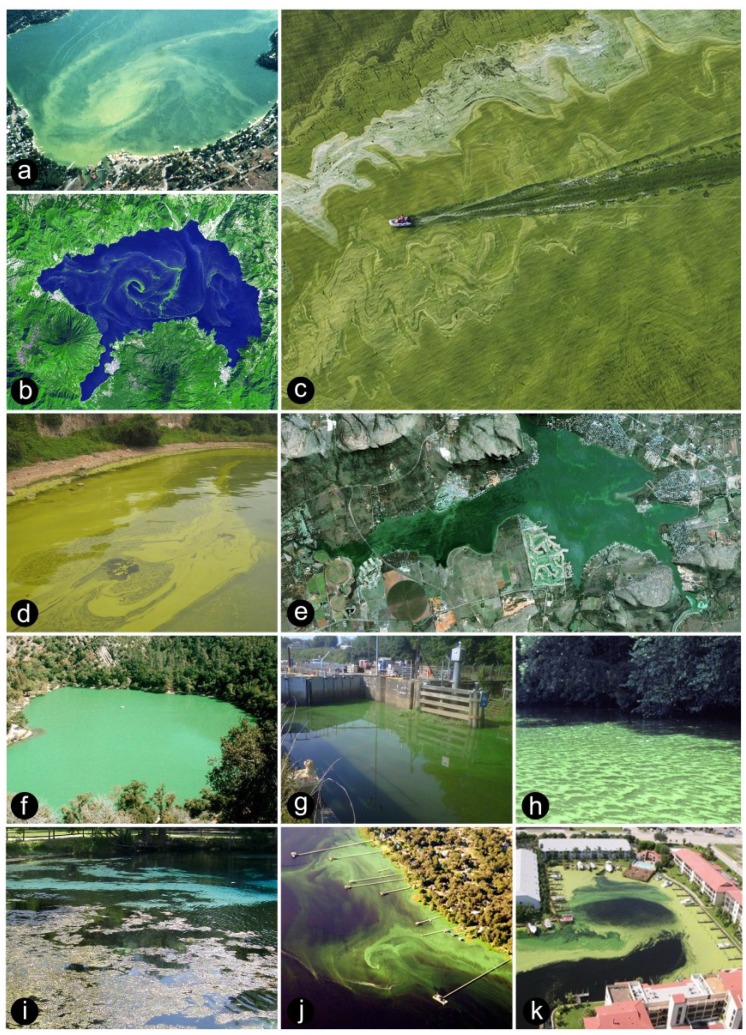
Cyanobacterial blooms, viewed for space and in the field. (**a**) Mixed *Microcystis* and *Anabaena* bloom in Liberty Lake, Washington, USA (Photo, Liberty Lake Sewer and Water District); (**b**) ASTER-TERRA image of a *Lyngbya* sp. Bloom in Lake Atitlan, Guatamala (Courtesy NASA); (**c**) Aerial view of *Microcystis*-dominated bloom in Lake Erie, USA (Photo, courtesy Peter Essick); (**d**) View of a *Microcystis*-dominated bloom in Meiliang Bay, Lake Taihu (Photo, Hans Paerl); (**e**) Satellite view of a cyanobacterial bloom in Hartbeespoort Dam Reservoir, South Africa, circa 2004 (Image^©^ 2014 Google, Digital Globe); (**f**) Mixed *Microcystis* and *Anabaena* bloom in Zaca Lake, California, USA in summer 1989 (Photo, Orlando Sarnelle); (**g**) A *Microcystis* bloom on the Cape Fear River, NC in front of “lock and dam No. 1” in 2013 (Photo, courtesy North Carolina Department of Environment and Natural Resources, Division of Water Resources); (**h**) *Microcystis* bloom on the Neuse River, North Carolina, circa 1983 (Photo, Hans Paerl); (**i**) A benthic *Lyngbya* sp. bloom floating to the surface of Weeki Watchee Springs, Florida, USA during summer 2003 (Photo, Hans Paerl); (**j**) Aircraft view of an *Anabaena* bloom on the St. Johns River, Florida, USA (Photo, courtesy Bill Yates/CYPIX); (**k**) Mixed *Microcystis* and *Anabaena* bloom at a development near the Indian River Lagoon, Florida, USA (Photo, courtesy John Burns).

The most obvious and troublesome sign of their contemporary ecological success is increasingly frequent and highly visible harmful cyanobacterial blooms, or CyanoHABs, in aquatic environments that have experienced recent human and climatic alterations; including nutrient over-enrichment (eutrophication), hydrologic alterations due to water withdrawal (for drinking, irrigation, industrial use) from streams, rivers and lakes, dams/reservoirs, construction of artificial waterways, lagoons and harbors, and alterations of a variety of benthic and planktonic habitats (Figure 1). CyanoHABs are considered harmful from an environmental perspective because they cause a loss of water clarity, which suppresses growth of aquatic macrophytes, with negative effects on invertebrate and fish habitats. Surface-dwelling blooms also negatively affect growth of sub-surface, more desirable (from food web and faunal health perspectives) eukaryotic phytoplankton taxa (e.g., diatoms, chlorophytes, flagellates) (Figure 1). Bacterial decomposition of dying blooms promotes oxygen depletion (hypoxia and anoxia), and subsequent fish kills. Lastly, many CyanoHABs produce secondary metabolites which can cause taste and odor problems in affected waters and lead to serious, acute intoxication in mammals (including humans) affecting the hepatopancreatic, digestive, endocrine, dermal and nervous systems [4] (Table 1). Overall, CyanoHABs threaten the ecological integrity and sustainability of aquatic ecosystems providing drinking water, irrigation, fishing and recreational resources.

**Table 1:** Most common cyanobacterial toxins known to negatively impact aquatic biota and consumers, including man. Shown are the toxin types, methods currently used for detection/quantification, and CyanoHAB genera known to produce toxins. Table adapted from Paerl and Otten [5].

Toxin	Detection Method(s) *	CyanoHAB Genera
Aeruginosin	HPLC, MS	*Microcystis*, *Planktothrix*
Anatoxin-a /Homoanatoxin-a	ELISA, HPLC, MS	*Anabaena*, *Aphanizomenon*, *Cylindrospermopsis*, *Lyngbya*, *Oscillatoria*, *Phormidium*, *Planktothrix*, *Raphidiopsis*, *Woronichinia*
Anatoxin-a(S)	AEIA, MS	*Anabaena*
Aplysiatoxins	MS	*Lyngbya*, *Oscillatoria*, *Schizothrix*
beta-Methylamino-L-alanine (BMAA)	ELISA, HPLC, MS	*Anabaena*, *Aphanizomenon*, *Calothrix*, *Cylindrospermopsis*, *Lyngbya*, *Microcysti*s, *Nostoc*, *Nodularia*, *Planktothrix*, *Phormidium*, *Prochlorococcus*, *Scytonema*, *Synechococcus*, *Trichodesmium*
Cyanopeptolin	HPLC, MS	*Anabaena*, *Microcystis*, *Planktothrix*
Cylindrospermopsin	ELISA, HPLC, MS	*Anabaena*, *Aphanizomenon*, *Cylindrospermopsis*, *Oscillatoria, Raphidiopsis*, *Umezakia*
Jamaicamides	MS	*Lyngbya*
Lyngbyatoxin	HPLC, MS	*Lyngbya*
Microcystin	ELISA, HPLC, MS, PPIA	*Anabaena*, *Anabaenopsis*, *Aphanizomenon*, *Aphanocapsa*, *Cylindrospermopsis*, *Gloeotrichia*, *Hapalosiphon*, *Microcystis*, *Nostoc*, *Oscillatoria*, *Phormidium*, *Planktothrix*, *Pseudoanabaena*, *Synechococcus*, *Woronochinia*
Nodularin	ELISA, HPLC, MS, PPIA	*Nodularia*
Saxitoxin	ELISA, HPLC, MS	*Anabaena*, *Aphanizomenon*, *Cylindrospermopsis*, *Lyngbya*, *Oscillatoria*, *Planktothrix*

* AEIA: acetylcholine esterase inhibition assay; ELISA: enzyme-linked immunosorbent assay; HPLC: high performance liquid chromatography; MS: mass spectrometry; PPIA: protein phosphatase inhibition assay.

Recurring blooms are commonplace in numerous nutrient-enriched lakes, reservoirs, riverine and estuarine systems, and they are expanding and thriving in some of the world’s largest inland freshwater ecosystems, including: Lake Victoria (Africa), Lake Erie and Lake Michigan (US-Canada), Lake Okeechobee (Florida, USA), Lake Ponchartrain (Louisiana, USA), Lake Taihu (China), and estuarine and coastal waters, e.g., the Baltic Sea, Caspian Sea, tributaries of Chesapeake Bay, North Carolina’s Albemarle-Pamlico Sound, Florida Bay, the Swan River Estuary in Australia, the Patos Lagoon (Brazil) and other coastal lagoonal estuaries in South America [4]; all of which are increasingly impacted by human nutrient enrichment, hydrologic modifications and climatic changes (heat waves, increasingly severe storms, droughts and floods).

Key to the broad distributions and overall ecological success of CyanoHABs are numerous adaptations to climatic extremes, including the formation of heat and desiccation-tolerant resting cells, or akinetes, cysts, the presence of photoprotective and desiccation-resistant sheaths and capsules, a wide array of photoprotective (including UV protective) cellular pigments, the ability to glide and (in planktonic environments) regulate buoyancy in order to adjust and optimize their position in the water column in response to irradiance and nutrient gradients [2,6]. They have also developed a wide array of physiological adaptations to periodic nutrient deplete conditions, including the ability to convert or “fix” atmospheric nitrogen (N_2_) into biologically-available ammonia [7], sequester (by chelation) iron [8], store phosphorus, nitrogen and other essential nutrients [6,9], and produce metabolites that enhance their ability to counter potentially adverse conditions in their immediate environment, including photooxidation, and yet to be discovered protective and adaptive functions [5,10,11,12]. Cyanobacteria have a diverse suite of mutualistic and symbiotic associations with prokaryotic and eukaryotic microbes, plants and animals that help ensure their (as well as their partners’) survival in environments too hostile for individual members to survive in [13,14].

This contribution will focus on specific aspects of cyanobacterial eco-physiology, habitat conditions and community composition and function, with the goal of gaining clues from these adaptive features and using them to develop mitigation strategies aimed at controlling CyanoHABs in aquatic ecosystems that favor their development and proliferation.

## 2. Cyanobacterial Bloom Taxa

**Figure 2:**
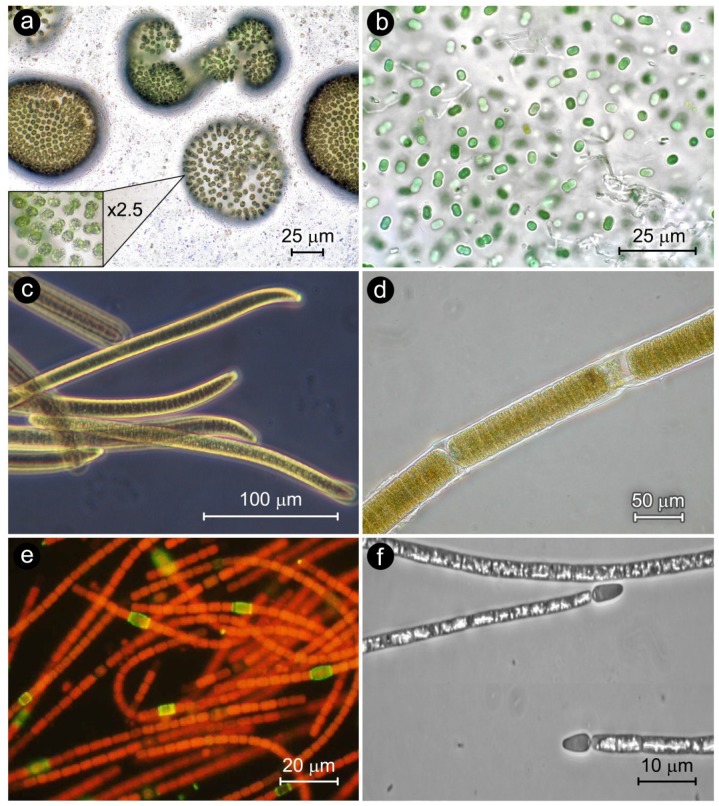
Photomicrographs of genera representing the three major cyanobacterial morphological groups. These include representative coccoid (a,b), filamentous non-heterocystous (c,d) and filamentous heterocystous (e,f) CyanoHAB genera. (**a**) *Microcystis* spp. (Photo, John Wehr); (**b**) *Synechococcus* sp. (Photo, Chris Carter). (**c**) *Oscillatoria* sp. (Photo, Hans Paerl); (**d**) *Lyngbya* sp. (Photo, Hans Paerl); (**e**) *Anabaena oscillarioides* immunofluorescence micrograph, showing the nitrogen-fixing heterocysts (green) (Photo, Hans Paerl); (**f**) *Cylindrospermopsis raciborskii* (Photo, Hans Paerl).

Cyanobacterial bloom taxa exist in three major morphologically-distinct groups (Figure 2). These include: (1) Coccoid cells, ranging from solitary (e.g., *Synechococcus*, *Chroococcus*) (<3 μm diameter), largely non-N_2_ fixing coccoid to ovoid cyanobacterial genera, which account for an important, and at times, dominant (>50%), fraction of freshwater, estuarine and marine phytoplankton biomass [15,16,17]. Other coccoid forms are aggregated in colonies that are widespread and sometimes dominate as blooms (e.g., *Microcystis*) in planktonic and benthic environments over a wide range of trophic states (ultraoligotrophic to hypertrophic) (Figure 2). Most of these genera do not fix nitrogen, and hence are dependent on combined nitrogen supplies for supporting growth. Some genera (e.g., *Microcystis*) can produce secondary metabolites that are toxic to inhabitants and consumers, ranging from zooplankton to fish to humans; (2) Filaments of mostly undifferentiated cells. This group is mostly comprised of non-N_2_ fixing genera (e.g., *Oscillatoria*, *Planktothrix*); however, some N_2_ fixing genera also exists (e.g., *Lyngbya*, *Trichodesmium*), and these genera can at times dominate, as blooms, in benthic and planktonic environments; (3) Filaments with highly differentiated, biochemically-specialized N_2_ fixing cells called heterocysts [18]. Heterocystous cyanobacteria are considered morphologically advanced because heterocysts appear to be an adaptation to ambient oxygen-rich conditions [18], which the cyanobacteria brought about during their proliferation on Earth. There are numerous bloom-forming genera in this group (e.g., *Anabaena*, *Aphanizomenon*, *Cylindrospermopsis*, *Nodularia*). In addition to planktonic bloom formers, benthic filamentous genera (*Calothrix*, *Rivularia*, *Scytonema*, *Lyngbya*, *Oscillatoria*) can undergo explosive growths as epiphytes, mats and biofilms (Figure 1). Filamentous and coccoid genera are also capable of producing secondary metabolites that can be toxic to a variety of animal consumers, ranging from zooplankton to fish to mammals, including humans [4,5,10,19,20].

## 3. Managing Cyanobacterial Blooms in a Human and Climatically-Altered World

**Figure 3:**
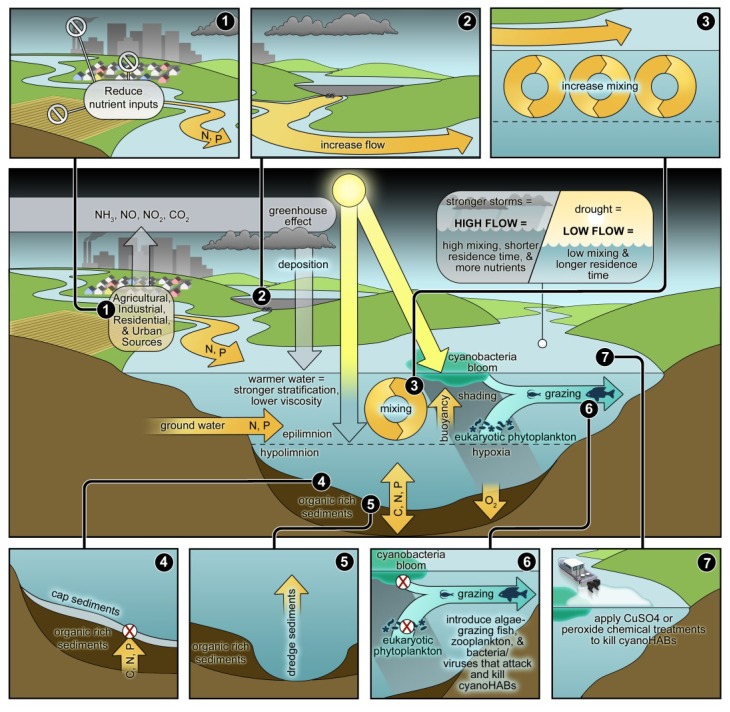
Conceptual diagram illustrating the various external and internal environmental and ecological factors controlling growth, accumulation (as blooms) and fate of cyanobacteria in aquatic ecosystems. Factors can act individually or in combined (synergistic, antagonistic) ways. They include surface and subsurface as well as atmospheric nutrient inputs, physical controls, including mixing/circulation, freshwater inputs and flushing (*i.e.*, residence time), light, temperature (including greenhouse gas mediated warming), grazing, and numerous within-system feedbacks, such as stratification and organic matter driven hypoxia, nutrient regeneration and light shading by blooms of subsurface phytoplankton populations. Lastly, physical forcing such as wind-driven vertical mixing, can lead to sediment resuspension, which will impact light and nutrient availability. Also shown are the various mitigation techniques and strategies for controlling/eliminating CyanoHABs, based on manipulating their ecological requirements and interfering with their growth strategies.

The frequencies, magnitudes and duration of CyanoHABs are controlled by a complex set of human- and climatically-driven factors in aquatic ecosystems (Figure 3). This presents a formidable challenge to water quality managers because at the ecosystem level, these physical, chemical and biotic drivers often co-occur, interact synergistically and/or antagonistically to control the activities (N_2_ fixation, photosynthesis) and growth of CyanoHABs [11,21] (Figure 3). How do we best address this challenge?

### 3.1. Nutrient Input Controls

Nutrient input reductions are obvious targets, because they have been shown to be effective in improving water quality, they can frequently be manipulated and hence should be a central part of any CyanoHAB mitigation strategy (Figure 3). We have long been aware that P input reduction is an effective means of reducing cyanobacterial dominance in aquatic, and especially freshwater, ecosystems (*c.f.*, Smith and Schindler [22]). However, there are increasing instances where N input reductions are also needed. This is especially the case in eutrophic, CyanoHAB susceptible, lakes, rivers, estuaries and coastal waters that are capable of assimilating more N and increasing their trophic state [23,24,25]. A key management priority is establishing N and P input thresholds (e.g., US EPA’s Total Maximum Daily Loads; TMDLs), below which CyanoHABs can be controlled in terms of magnitude, temporal and spatial coverage [24]. The ratios of N to P inputs should be considered when developing these thresholds. Ideal input ratios are those that do not favor CyanoHAB species over more desirable taxa, but there does not appear to be a universal ratio—above or below—which CyanoHABs or their toxin production can be consistently and reliably controlled [26]. In addition, there is quite a range of ecosystem sensitivities to CyanoHAB development and persistence; sensitivity depends on system size and morphometry, mixing depths and intensities, flushing rates/water residence times. For these reasons, total nutrient loads and concentrations need to be considered on an ecosystem case basis in CyanoHAB management [23,26]. It is generally thought that total molar N:P ratios above ~15 discourage CyanoHAB dominance [22,27]. However, if the nutrient load and internal concentrations of N or P are extremely high (*i.e.*, above saturation levels), a ratio approach for CyanoHAB control is not likely to be effective [4,26].

There are many ways to reduce nutrient inputs on the ecosystem scale [22,28]. Nutrient inputs have been classified as point source and non-point source. Point sources are often associated with well-defined and identifiable discharge sites. Targeting point sources is often attractive, because they can account for a highly significant share of P and N loading, they are readily accessible, and hence from a regulatory perspective, easiest to control. The major challenge that remains in many watersheds is controlling more diffuse non-point sources, which often constitute the largest sources of nutrients; hence, their controls are likely to play a critical role in mitigating CyanoHABs in the context of human and climatically-driven environmental changes currently taking place.

### 3.2. Phosphorus Management

Phosphorus inputs are dominated by: (1) non-point source surface runoff; and (2) point sources such as effluents from wastewater treatment plants, industrial and municipal discharges; and (3) subsurface drainage from septic systems and groundwater. Among these, point sources are often the focus of P reductions. In agricultural and urban watersheds, non-point surface and subsurface P inputs are of increasing concern. Increased P fertilizer use, generation and discharge of animal waste, soil disturbance and erosion (*i.e.*, sedimentation), conversion of forests and grasslands to row-crop and other intensive farming operations, and the proliferation of septic systems accompanying human population growth are rapidly increasing non-point P loading [29]. In agricultural and urban watersheds, non-point sources can account for >50% of annual P loads [29]. Because of the diffuse nature of these loadings, they are often difficult to identify and address. They are also very susceptible to mobilization due to an increase in episodic rainfall events, major storms and tropical cyclones.

The manner in which P is discharged to P-sensitive waters plays a role in CyanoHAB proliferation and management. Considerations include: (1) total annual (*i.e.*, chronic) P loading; (2) shorter-term seasonal and event-based pulse (*i.e.*, acute) P loadings; (3) particulate *vs.* dissolved P loading; and (4) inorganic *vs.* organic P loading; and (5) hydrologic (rainfall and freshwater discharge) variability. In terms of overall ecosystem P budgets and long-term responses to P loadings (and reductions), annual P inputs are of fundamental importance. When and where P enrichment occurs can determine the difference between bloom-plagued *vs.* bloom-free conditions. For example, if a large spring P discharge event precedes a summer of dry, low flow conditions in a relatively long residence time water body, the spring P load will most likely be available to support summer bloom development and persistence. Effective exchange and cycling between the water column and bottom sediments can retard P transport and hence retain P [30] (Figure 3). As a result, acute P inputs due to high flow events may be retained longer than would be estimated based on water flushing time alone. As such, water bodies exhibit both rapid biological responses to and a “memory” for acute P loads.

Unlike N, which can exist in dissolved gaseous forms, P exists only in dissolved ionic and particulate forms in natural waters. Therefore, the focus is on dissolved *vs.* particulate forms of inorganic and organic P. Dissolved inorganic P (DIP) exists as orthophosphate (PO_4_^3−^), which is readily assimilated by all CyanoHAB taxa. Cyanobacteria can accumulate and store assimilated P intracellularly as polyphosphates; which can be available for subsequent use during times of P depletion [9]. Dissolved organic P (DOP) can also be a significant fraction of the total dissolved P pool. DOP can be assimilated by bacteria, microalgae and cyanobacteria, although not as rapidly as PO_4_^3−^ [31]. A large fraction of the assimilated DOP is microbially-recycled to DIP, enhancing P availability. The role of particulate P (as inorganic or organic forms) in aquatic production and nutrient cycling dynamics is less well understood. Particulate P (PP) may provide a source of DIP and DOP via desorption and leaching, and it may serve as a “slow release” source of DIP. In this manner, a fraction of PP can serve as a source of biologically-available P and hence play a role in CyanoHAB control. On the ecosystem-scale, sedimented PP serves as an important source of stored P for subsequent release, especially during hypoxic/anoxic periods. It is therefore essential to include both dissolved and particulate P when managing P inputs, especially under hydrologically-variable conditions accompanying climate change.

### 3.3. Nitrogen Management

Nitrogen exists in multiple dissolved, particulate and gaseous forms. Many of these forms are biologically-available and readily exchanged within and between the water column and sediments [32,33]. In addition, biological nitrogen (N_2_) fixation and denitrification control the exchange between inert gaseous atmospheric N_2_ and biologically-available combined N forms. Combined forms of N include dissolved inorganic N (DIN; including ammonium (NH_4_^+^), nitrate (NO_3_^−^) and nitrite (NO_3_^−^)), dissolved organic N (DON; e.g., amino acids and peptides, urea, organo-nitrates), and particulate organic N (PON; polypeptides, proteins, organic detritus). These forms can be supplied from non-point and point sources. Non-point sources include surface runoff, atmospheric deposition and groundwater, while point sources are dominated by municipal, agricultural and industrial wastewater. In rural and agricultural settings, non-point N inputs tend to dominate (>50% of total N loading), while in urban centers, point sources often dominate [28]. All sources contain diverse organic and inorganic N species in dissolved and particulate forms; representing a mixture of biologically-available DIN, DON and PON.

In contrast to P dynamics, where SRP is the main available form, N occurs in many available forms and redox states, and its biogeochemistry in natural waters is mediated mainly by biological processes. For example, as mentioned above, most N transported from watersheds occurs as oxidized nitrate, primarily due to microbial N transformations along the flowpath and the relatively high cost of nitrate assimilation *vs.* other N forms. N is most actively assimilated and recycled as NH4^+^ and organic forms by the microbial food web. Recycled N in reduced forms, such as NH4^+^ and urea, is a major source of N to CyanoHABs [34,35].

Nitrogen inputs are dynamic, reflecting land use, population and economic growth and hydrologic conditions [36]. The means and routes by which human N sources impact eutrophication are changing [28]. Among the most rapidly-growing (amount and geographic scale) sources of human N loading are surface runoff, groundwater and atmospheric deposition. Atmospheric N loading is also an often-overlooked and expanding source of N input to N-sensitive waters [28,37]. As with P, N input and cycling dynamics are sensitive to patterns and intensities of precipitation, freshwater flow, which controls mobilization in the watershed and discharge to N-sensitive waters. 

### 3.4. Rationale for Dual Nutrient (N and P) Controls

Phosphorus removal has helped improve water quality [22], but recent studies indicate that eutrophic systems supporting CyanoHABs often exhibit maximum algal growth in response to combined N and P additions, or at times only N additions, not just P additions [38,39,40,41]. These shifts in the freshwater nutrient limitation paradigm, resulting from increasing and uncontrolled nutrient inputs due to urbanization and expanding agriculture production [28,29,36], have important management implications.

Severe blooms of toxic, non-N_2_ fixing CyanoHABs (e.g., *Microcystis*) continue in nutrient-sensitive systems worldwide (Figure 1), despite improvements from P-focused control [42]. Because these taxa cannot fix atmospheric N_2_, they require combined N sources (ammonium, dissolved organic N, oxidized N) to support growth. Large increases in watershed and atmospheric N inputs have filled this requirement [28,29]. What common factors cause these patterns in freshwaters experiencing non-N_2_ fixing cyanobacteria blooms?

In addition to domestic wastewaters, non-point P and N from agricultural fertilizers exert a major strain on aquatic systems [28,29,33]. In CyanoHAB-plagued Lake Erie, USA, soluble reactive P has increased as a component of total P, indicating the increasing need to control non-point P sources [42]. The removal of N is difficult, because of its complex biogeochemical transformations and non-point origin. Control of non-point N sources has attracted limited attention in the freshwater eutrophication literature (*c.f.*, [22,43]).

The strong case made for non-point P reduction should be extended to include control of non-point N for effective long-term eutrophication control. Increased production and application of synthetic N fertilizers [36] has caused exponential increases in bioavailable N compounds to receiving waters [28]. These N increases coincide with new or reappearing eutrophication problems worldwide, in the form of toxic, non-N_2_ fixing algal blooms (e.g., *Microcystis*) [41].

**Figure 4:**
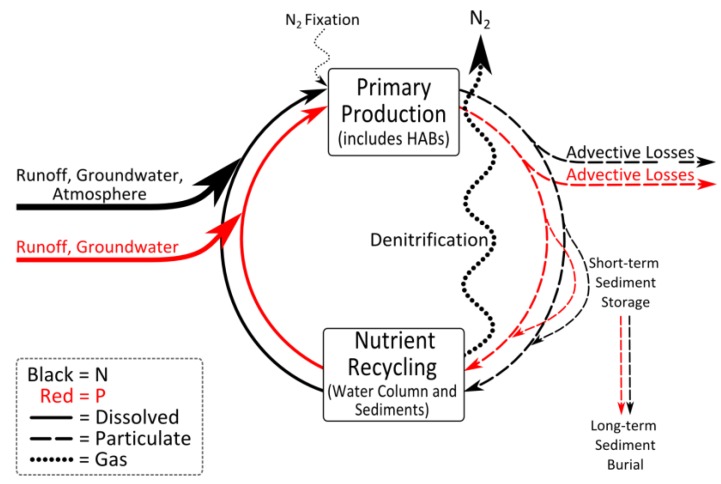
Conceptual diagram, illustrating nitrogen (N) and phosphorus (P) inputs, outputs and storage in aquatic ecosystems. Note that only N has gaseous forms (dotted lines) that can exchange with the atmosphere. In lakes that have had a history of excessive N and P loading, N limitation is perpetuated because there is more N “lost” via denitrification than gained by N_2_ fixation. This is the basis for recommending that both N and P inputs be controlled in eutrophic waters plagued by CyanoHABs.

Intuitively, increased N enrichment should enhance P-limitation and emphasize the need for P control in eutrophic waters. *A priori*, N_2_ fixation was assumed to supply ecosystem N needs as long as enough P is available because some cyanobacteria and bacteria can fix atmospheric N_2_ into biologically available ammonium [44]. However, empirical data and modeling show that N_2_ fixation cannot sustain ecosystem N-demand [23,45]. This is because N_2_ fixation is controlled by environmental factors beyond phosphorus availability [46]. External N inputs can help satisfy ecosystem N-demand and accelerate eutrophication [38,43,47,48]. Nitrogen-enhanced algal production increases the organic matter load, which stimulates sediment and water column respiration and nutrient regeneration, making N and P more available to further enhance primary production and CyanoHABs (Figure 4).

Because N occurs in gaseous forms, while P has no gaseous form during internal cycling, P inputs tend to be retained, while N inputs are “lost” to the atmosphere, with denitrification being a major loss mechanism in eutrophic systems [24,26]. Biogeochemical processes thus enhance retention of P relative to N and perpetuate N limitation. Increasing human N loading helps alleviate N limitation, but it also leads to accelerated eutrophication and CyanoHAB proliferation. That is a major reason why human N inputs need to be constrained (along with P inputs). High N loads can also interfere with P burial in sediments by affecting the sulfur cycle, resulting in enhanced internal P loading [49]. This positive feedback loop may cause the resurgence of algal blooms in eutrophic lakes from previous loadings of N and P stored in the sediments (Figure 4). It is concluded that the freshwater “P-only” management paradigm should be shifted to one incorporating N-driven eutrophication in lakes with suitable physical and chemical conditions (e.g., seasonally warm temperatures, shallow photic zones, excessive nutrient inputs; Figure 1) for CyanoHAB development.

## 4. The Interactions of Physical, Biological and Nutrient Controls of Cyanobacteria in a Climatically-Changing World

### 4.1. Climate Change: Its Role in Modulating CyanoHABs

**Figure 5:**
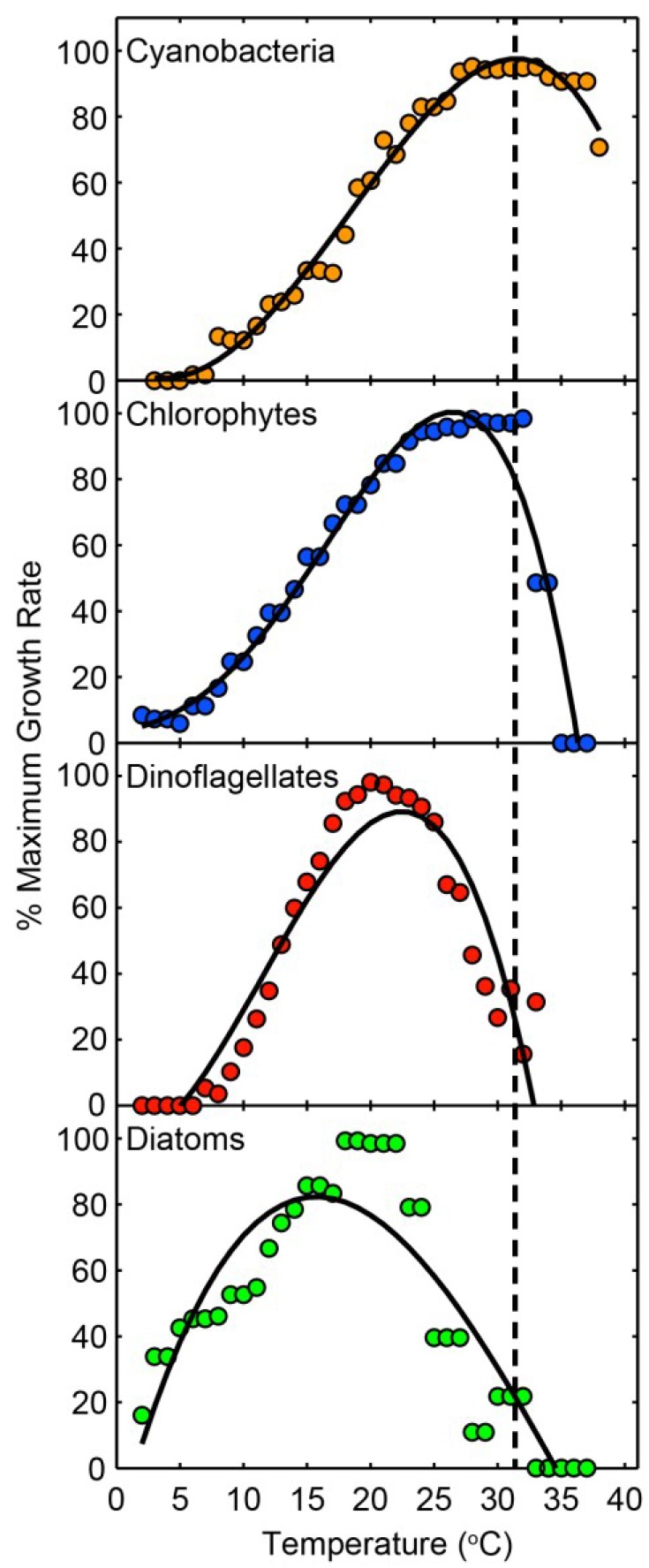
Temperature-growth relationships among four different taxonomic groups (cyanobacteria, chlorophytes, dinoflagellates and diatoms). The dashed line is for comparison of optimal cyanobacterial growth temperature with temperature-growth relationships in other groups. Data points are 5 °C running bin averages of percent maximum growth rates from 3–4 species within each group. Fitted lines are third order polynomials and are included to emphasize the shape of the growth *vs.* temperature relationship. Data sources and percent maximum growth rates of individual species are provided in [41].

While there is a rich literature showing a clear link between nutrient (N, P, trace metals) availability and the composition, distribution and abundance of cyanobacterial taxa in aquatic ecosystems [2,50,51], climate change plays an interactive modulating role. Rising global temperatures, altered precipitation patterns and changes in hydrologic properties (*i.e.,* freshwater discharge or flushing rates) of water bodies strongly influence growth rates, composition and bloom dynamics of cyanobacteria [41,48,52,53,54,55,56]. Warmer temperatures favor surface bloom-forming cyanobacterial genera because as prokaryotes, they tend to show a strong preference for relatively warm conditions, and their maximal growth rates occur at relatively high temperatures; often in excess of 25 °C [57,58,59]. At elevated temperatures, cyanobacteria can often outcompete eukaryotic algae ‎ [60,61]. Specifically, as the growth rates of the eukaryotic taxa reach their maxima or decline in response to warming, cyanobacterial growth rates reach their optima (Figure 5).

Warmer surface waters also promote more intense vertical stratification. The strength of vertical stratification depends on the density difference between the relatively warm surface layer and the colder, deeper waters. In marine systems, salinity gradients also induce stratification. As mean temperatures rise, waters will begin to stratify earlier in the spring, and stratification will persist longer into the fall [62,63,64,65,66]. Northern lakes, rivers and estuarine ecosystems have shown warming of surface waters, leading to earlier “ice out” and later “ice on” periods and stronger vertical temperature stratification [62,63]. This has extended the periodicity and range of cyanobacterial species, especially bloom-forming ones. Evidence for this can be obtained from lakes in northern Europe and North America, some of which no longer have ice on them during winter months [65,‎66].

In polar regions, even small increases in warming can significantly impact cyanobacterial activity and dominance. Along the margins of the Antarctic continent, cyanobacteria often thrive on exposed soils, glaciers, ice shelves, frozen lakes and stream beds, where they can comprise most of the ecosystem biomass [67]. Virtually all the photosynthetic production, nutrient cycling and trophic transfer are confined to this ice-free period [67,68,69]. Global warming will likely have a positive effect on the cyanobacterial communities by providing an extended window of liquid water conditions. Polar cyanobacteria are also highly responsive to increasing temperatures [70,71,72].

Increasing concentration of atmospheric CO_2_ from growing fossil fuel emissions, while being a key driver of global warming (*i.e.*, greenhouse effect) and cyanobacterial growth potential, may also benefit CyanoHABs by helping relieve carbon (CO_2_) limitation, especially during active growth periods when CO_2_ demands are high [72,73]. Another symptom of climatic changes potentially impacting cyanobacterial communities is increasing variability and more extremeness in precipitation amounts and patterns. Storm events, including tropical cyclones, nor’easters, and summer thunderstorms, are becoming more extreme, and have higher amounts and intensities of rainfall [74,75,76,77,78,79]. Conversely, droughts are becoming more severe and protracted [80]. These events cause large changes in hydrologic variability, *i.e.*, wetter wet periods and drier dry periods. This has led to more episodic “flashy” discharge periods in which large amounts of nutrients are captured and transported in runoff events leading to rapid and profound nutrient enrichment of receiving waters. If such events are followed by periods of extended drought in which freshwater flow decreases dramatically and residence time of receiving waters increases, conditions favoring cyanobacterial dominance and bloom formation will greatly improve. This will be particularly effective if it is accompanied by warming, since as a phytoplankton group, cyanobacteria have relatively slow growth rates at moderate temperatures [59], which would increase in a warmer regime [55,81]. The combination of episodic loads of nutrients (e.g., spring runoff period), followed by a protracted warm (summer), low discharge period (long residence time) can promote cyanobacterial growth and bloom potentials in geographically-diverse regions [41].

Higher amounts of freshwater runoff can enhance vertical density stratification (reduced vertical mixing) in waters having appreciable salinity, including estuarine and coastal waters as well as saline lakes and rivers; by allowing relatively light freshwater lenses to establish themselves on top of heavier (denser) saltwater. The resultant enhanced vertical stratification will favor phytoplankton capable of vertical migration to position themselves at physically-chemically favorable depths [54]. Bloom-forming cyanobacteria are capable of rapidly altering their buoyancy in response to varying light, temperature and nutrient regimes, by periodically forming blooms in surface waters [82]. Surface blooms are inhospitable to grazers and eukaryotic taxa that cannot handle the excessive irradiance in these waters. Many bloom taxa have photoprotective pigments, enabling them to persist as surface blooms [83], while sub-surface algal taxa will be shaded by surface blooms, leaving them in sub-optimal light conditions (*c.f.*, Figure 3).

More extensive summer droughts, rising sea levels, and increased use of freshwater for irrigation, industrial and drinking water use can lead to salinization, which has increased worldwide. Some CyanoHAB genera are salt-tolerant to varying degrees, even though they are most common to freshwater ecosystems, because these waters are nutrient-enriched. These genera include the N_2_ fixers *Anabaena*, *Anabaenopsis*, *Aphanizomenon*, *Nodularia*, and some species of *Lyngbya* and *Oscillatoria*, as well as non-N_2_ fixing genera, including *Microcystis*, *Oscillatoria*, *Phormidium* and picoplanktonic genera (*Synechococcus*, *Chroococcus*). Some strains of *Microcystis aeruginosa* can tolerate salinities of up to 10, nearing 30% of seawater salinity [84], and in Patos Lagoon, Brazil, they can thrive under “mixohaline” conditions (Carstensen *et al.*, in prep.). Some *Anabaena* and *Anabaenopsis* species can withstand salinities up to 15 [85], while the Baltic Sea bloom-former *Nodularia spumigena* can tolerate salinities exceeding 20 [86,87].

The filamentous, bloom-forming and toxin-producing diazotroph *Cylindrospermopsis raciborskii* has undergone recent expansion of its geographical range. Its expansion gained attention following an outbreak of a severe hepatitis-like disease on Palm Island (Australia), the so-called “Palm Island mystery disease” [‎4]. This outbreak followed treatment of a local water supply reservoir in Australia with copper sulfate. Epidemiological studies confirmed the linkage between the “mystery disease” and the newfound presence of *Cylindrospermopsis* [4]. Lysis of the *Cylindrospermopsis* bloom released the highly stable toxin cylindrospermopsin into the water supply.

*Cylindrospermopsis* has been described as a tropical/subtropical genus [88]. However, *C. raciborskii* was documented in Europe during the 1930s, and showed a progressive colonization from Greece and Hungary towards higher latitudes near the end of the 20th century [88]. It was described in France in 1994, in The Netherlands in 1999, and it is now widespread in lakes in northern Germany [62,‎65], and has also been detected in Canada [89]. *C. raciborskii* was noted in Florida almost 35 years ago, after which it aggressively proliferated throughout eutrophic lakes and rivers [90]. It is now present throughout the US in reservoirs, lakes, rivers and even oligohaline estuarine waters experiencing various degrees of eutrophication and loss of water clarity [51,91]. This combination is significant because *C. raciborskii* is adapted to low light conditions (high turbidity) typifying eutrophic waters. It also prefers water temperatures above 20 °C, and survives adverse conditions using specialized vegetative resting cells (akinetes). This scenario hints of a link to eutrophication *and* global warming. The activation of akinetes in this and other heterocystous species (e.g., *Aphanizomenon ovalisporum*) is strongly temperature regulated [92]. Increases in ambient temperatures may therefore play an important role in the geographic dispersal strategy, and potential expansion of this and other akinete-forming genera (*Anabaena*, *Anabaenopsis*, *Nodularia*).

Blooms of the filamentous, non-heterocystous, toxin-producing bloom former *Lyngbya* spp. have become increasingly common and problematic in nutrient-enriched freshwater and marine ecosystems; including those that have experienced human disturbances such as dredging, municipal waste inputs and the discharge of nutrient-laden agricultural runoff [93,94]. *Lyngbya* is a ubiquitous genus, with various species occurring in planktonic and benthic habitats. *L. majuscula* (marine-benthic) and *L. wollei* (freshwater-benthic and planktonic) are opportunistic invaders. Following large climatic and hydrologic perturbations such as tropical cyclones, *L. wollei* has proven to be an aggressive initial colonizer of perturbed systems [51,95]. *Lyngbya* blooms can proliferate as dense, attached or floating mats that shade other primary producers, which enable them to dominate the system by effectively outcompeting them for light (Figure 3). As is the case with *Cylindrospermopsis* and *Microcystis*, this genus benefits from both human and climate-induced environmental change.

While diverse field studies have demonstrated the expansion of cyanobacterial taxa in response to changing climatic conditions [88,89,90], in certain cases, such as altered hydrology, the drivers cannot be solely attributed to climate change, but rather reflect the complex interactions of human alteration of hydrology and changing rainfall patterns.

### 4.2. The Role of Physical Factors

Physical factors, including altering turbulence, vertical mixing and hydrologic flushing play key roles of cyanobacterial blooms in aquatic ecosystems. It is well known that vertical stability (thermal or salinity stratification), and long water replacement (residence or flushing) times favor cyanobacteria over eukaryotic phytoplankton; hence, disruption of these conditions can, under certain circumstances and in specific systems, modulate cyanobacterial bloom dynamics (Figure 3). Vertical mixing devices, bubblers and other means of breaking down destratification have proven effective in controlling CyanoHABs in relatively small impoundments such as farm and fish ponds [12,96]. However, these devices have limited applicability in large lake, estuarine and coastal waters, because they cannot exert their forces over such large areas and volumes.

**Figure 6:**
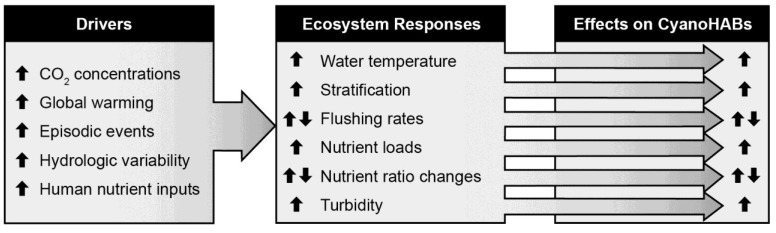
Linkage of major anthropogenic and climatic environmental drivers of ecosystem change to their impacts on CyanoHAB potentials.

Increasing the flushing rates, and thereby decreasing water residence time (or water age), can be effective in reducing or controlling bloom taxa; mainly because cyanobacteria exhibit relatively slow growth rates, relative to eukaryotes. Horizontal flushing, by increasing the water flow through lakes or estuaries, can reduce the time for cyanobacterial bloom development [97] (Figure 6). While this approach can suppress CyanoHABs, hydrologic changes can be quite expensive, dependent on freshwater supplies for flushing, and restricted to relatively small water bodies.

Water quality managers must ensure that the flushing water is relatively low in nutrient content, so as not to worsen the enrichment problem, especially in large water bodies, which tend to have a long “memory” for nutrient inputs. For example, in hypereutrophic Lake Taihu, China, efforts to reduce *Microcystis*-dominated blooms by flushing this large lake with nearby Yangtze River water, which reduced the lake’s overall residence time from ~1 year to ~200 days, have not had a significant impact on reducing blooms. Yangtze River water is exceedingly high in dissolved N and P compounds, making it a nutrient source for further eutrophication. Furthermore, the inflow pattern of Yangtze River water has altered the circulation regime of Taihu, and entrained or “trapped” blooms in the lake’s northern bays, where they are most intense to begin with [98]. Lastly, few catchments have the luxury of being able to use precious water resources normally reserved for drinking or irrigation for flushing purposes. This is especially true of regions susceptible to extensive droughts (e.g., Australia, western USA).

### 4.3. Non-Nutrient Chemical Controls

Chemical treatments have been used to control cyanobacterial blooms. These include the applications of algaecides, the most common of which is copper sulfate. Copper sulfate is effective, but it can be toxic to a wide variety of plant and animal species and its residue in the sediments is problematic as a legacy pollutant. More recently, hydrogen peroxide has been shown to be an effective algaecide [99]. It is an attractive alternative to copper sulfate because it is selective for cyanobacteria (*vs.* eukaryotic algae and higher plants), and poses no serious long-term pollution problem. Furthermore, oxidation by peroxide is stimulated by light and causes breakdown of microcystins into peptide residues, and hence may serve to detoxify waters impacted by microcystin-producing blooms. Both of these treatments are restricted to fairly small impoundments. Caution must be practiced with using algaecides in waters affected by toxin-producing CyanoHABs. For example, copper sulfate, while killing CyanoHABs, will also lead to release of their endotoxins into the bulk water phase, causing problems with drinking and irrigation water supplies [4].

An alternative (to algaecides) chemical approach is to employ precipitation of P, thereby keeping it “locked up” in the sediments (Figure 3). Traditionally, alum, gypsum and lime have been used to precipitate P, but treatments with these agents often do not lead to permanent sediment retention and burial of P. An alternative treatment, called “Phoslock” uses a bentonite clay infused with the rare earth element lanthanum [100]. The lanthanum ions are electrostatically bound to the bentonite, while also strongly binding to phosphate anions. The bound phosphate then settles out of the water column and the thin layer (~1 mm) of Phoslock on the sediment surface forms a barrier to phosphate diffusing out of the sediments [100]. Phoslock has been shown to be reasonably effective in small reservoirs, where it can lead to P-limited conditions that can control CyanoHAB production [100]. Additionally, the thin Phoslock layer increases the critical erosional velocity of fine-grained surficial sediments, which should reduce the frequency of resuspension events and associated pulse nutrient loading (although, this will largely be effective in relatively small, deep lakes). Sediment stabilization and reduced phytoplankton biomass may also aid restoration of macrophyte communities in shallow, eutrophic systems where light limitation and low root anchoring capacity of fine-grained, organic-rich sediments often synergistically determine CyanoHAB dominance. Shallow lakes in which wind-driven sediment resuspension is a common feature are not good candidates for precipitation techniques like Phoslock.

### 4.4. Manipulating Sediments

Even when external nutrient inputs are reduced, the legacy of eutrophication in sediments can perpetuate high internal nutrient loads and provide a steady inoculum of algal spores or cysts that can continue to fuel CyanoHABs. Therefore, either removing sediments or capping them so that sediment-water column exchange of nutrients and algal cells is restricted has been used to control CyanoHABs (Figure 3).

Sediment removal involves expensive dredging, disturbance of lake bottoms, which can lead to additional nutrient (and potentially toxic substances) release and destruction of benthic flora and fauna [101]. There are examples of successful eradication of CyanoHABs using this approach, e.g., Lake Trummen, Sweden, a small (~1 km^2^, mean depth 1.6 m) lake that experienced CyanoHAB related water quality degradation in response to domestic sewage and industrial nutrient inputs during the mid-1900s [101]. Suction dredging the upper 0.5 m of sediments during a 2-year period led to highly significant decreases in nutrient concentrations and CyanoHABs [101]. The Lake Trummen success can largely be attributed to its small, easily manipulatable size, and the ability to effectively target reductions of external nutrient loads from its small (13 km^2^) watershed, following dredging. In other sediment dredging efforts on sections of large lakes, beneficial results have not been achieved, largely because the legacy of nutrient contamination of sediments is far greater than can be removed (e.g., Lake Taihu, China; Lake Okeechobee, USA). A serious logistic challenge with dredging is that the sediments removed from a water body must be exported and deposited out of the drainage basin, in order to avoid sediment-associated nutrients from leaching back into the system.

### 4.5. Biological Controls

Biological controls include a number of approaches to change the aquatic food web to increase grazing pressure on cyanobacteria or to reduce recycling of nutrients. Biomanipulation approaches can include introducing fish and benthic filter feeders capable of consuming cyanobacteria, or introduction of lytic bacteria and viruses. The most common biomanipulation approaches are intended to increase the abundance of herbivorous zooplankton by removing zooplanktivorous fish or introducing piscivorous fish (Figure 3). Alternatively, removal of benthivorous fish can reduce resuspension of nutrients from the bottom sediments. Questions have been raised about the long-term efficacy of curtailing cyanobacterial blooms by increasing grazing pressure, because this may lead to dominance by ungrazable or toxic strains [102,103,104]. Presently, biomanipulation is viewed as one component of an integrated approach to water quality management in circumstances in which nutrient reductions alone are insufficient to restore water quality [105,106,107,108,109]. Otherwise, nutrient management is the most practical, economically feasible, environmentally-friendly, long-term option.

## 5. Concluding Remarks and Recommendations

Cyanobacteria are globally distributed and their activities and relative roles in production and nutrient cycling dynamics are controlled by a complex set of environmental variables that are heavily influenced by human and climatic perturbations. Their long evolutionary history has enabled them to structurally and functionally diversify, which in turn has enabled them to adapt to short-term (*i.e.*, diel, seasonal, decadal) and longer term (geological) environmental perturbations and more gradual changes. Because they have experienced major and extreme climatic shifts over these time scales, they are well suited to deal with and take advantage of various climatic changes that we are now experiencing, including warming, altered rainfall patterns and amounts, resultant changes in freshwater runoff, flushing and vertical stratification.

In addition to climatically-driven environmental changes known to influence cyanobacterial growth and dominance, the most significant anthropogenically-influenced factors include: (1) nutrient (especially N and P) enrichment; (2) hydrological changes, including freshwater diversions, the construction of impoundments such as reservoirs, water use for irrigation, drinking, flood control, all of which affect water residence time or flushing rates; (3) biological alterations of aquatic ecosystems, including manipulations of grazers (from zooplankton to fish), and lastly (4) the discharge of xenobiotic compounds, e.g., heavy metals, herbicides and pesticides, industrial and domestic chemicals, antibiotics and other synthetic growth regulators, all of which affect phytoplankton community growth and composition (Figure 6).

Effective long term management of CyanoHABs using the above-mentioned controls must take into consideration the ecological and physiological adaptations that certain taxa possess to circumvent some controls. Examples include: (1) the ability of N_2_ fixing taxa to exploit N-limited conditions; (2) the ability of certain buoyant taxa to counteract mixing and other means of man-induced destratification aimed at minimizing cyanobacterial dominance; (3) specific mutualistic and symbiotic associations that cyanobacteria have with other microorganisms, which promote “internal” nutrient regeneration and can lead to bloom persistence and duration despite controls.

In an overwhelming number of cases, nutrient input reductions are the most direct, simple, and ecologically/economically feasible cyanoHAB management strategy; this is especially true for ecosystems experiencing effects of climate change, including warming and/or increased hydrologic variability and extremes. Nutrient input reductions that can decrease cyanobacterial competitive abilities, possibly combined with physical controls (in systems that are amenable to those controls) are often the most effective strategies. Nutrient (specifically N) treatment costs can be prohibitive however, in which case, alternative nutrient removal strategies may prove attractive; including construction of nutrient-stripping wetlands and the stimulation of macrophyte growth as a means to provide alternative nutrient sinks and reduce the eutrophication of water ways, stocking of herbivorous (and specifically cyanobacteria consuming) fish and shellfish species.

Water quality managers will have to accommodate the hydrological and physical-chemical effects of climatic change in their strategies. Given the competitive advantages (over eukaryotic algae) that cyanobacteria enjoy in a more climatically-extreme period we are now experiencing, efforts aimed at control and management of cyanobacteria will need to be flexible enough to incorporate this extremeness. For example, nutrient input reductions aimed at stemming eutrophication and cyanobacterial bloom potentials will need to be carefully gaged and potentially changed to accommodate higher cyanobacterial growth potentials due to warming and increasing bloom potentials due to stronger vertical stratification and positive nutrient cycling feedbacks.

Lastly, without a comprehensive strategy to reduce greenhouse gas emissions, future warming trends and their impacts on aquatic ecosystems will likely only lead to further expansion and dominance of these ecosystems by cyanobacteria.
